# The Role of PARP Inhibitors in the Treatment of Prostate Cancer: Recent Advances in Clinical Trials

**DOI:** 10.3390/biom11050722

**Published:** 2021-05-12

**Authors:** Mingyue Xia, Zhigang Guo, Zhigang Hu

**Affiliations:** Jiangsu Key Laboratory for Molecular and Medical Biotechnology, College of Life Sciences, Nanjing Normal University, 1 WenYuan Road, Nanjing 210023, China; xiaxia688@outlook.com

**Keywords:** PARP inhibitors, synthetic lethality, prostate cancer (PC), the latest research progress, combination strategies

## Abstract

Poly (adenosine diphosphate-ribose) polymerase inhibitors (PARPis) belong to a class of targeted drugs developed for the treatment of homologous recombination repair (HRR)-defective tumors. Preclinical and limited clinical data suggest that PARP inhibition is effective against prostate cancer (PC) in patients with HRR-deficient tumors and that PARPis can improve the mortality rate of PC in patients with *BRCA1/2* mutations through a synthetic lethality. Olaparib has been approved by the FDA for advanced ovarian and breast cancer with *BRCA* mutations, and as a maintenance therapy for ovarian cancer after platinum chemotherapy. PARPis are also a new and emerging clinical treatment for metastatic castration-resistant prostate cancer (mCRPC). Although PARPis have shown great efficacy, their widespread use is restricted by various factors, including drug resistance and the limited population who benefit from treatment. It is necessary to study the combination of PARPis and other therapeutic agents such as anti-hormone drugs, USP7 inhibitors, BET inhibitors, and immunotherapy. This article reviews the mechanism of PARP inhibition in the treatment of PC, the progress of clinical research, the mechanisms of drug resistance, and the strategies of combination treatments.

## 1. Introduction

Prostate cancer (PC) is a major cause of morbidity and mortality in men. It is the most common noncutaneous cancer in men, with an estimated 1.6 million cases and 0.36 million deaths annually [1]. For PC therapy, the initial treatment is androgen deprivation therapy (ADT), which “starves” PC cells by targeting their dependence on androgen receptor (AR) signal transduction. Taxanes and bilateral orchiectomy may also be used. However, there are significant differences in individual responses, and other treatment options are limited [2]. Following ADT [3], most hormone-sensitive patients progress to castration-resistant prostate cancer (CRPC), which is the leading cause of death. Patients with PC, especially CRPC, often have mutations in genes such as *BRCA1*, *BRCA2*, *ATM*, *CHEK2*, *RAD51D*, and *PALB2* [4,5,6,7].

Genomic DNA is continuously confronted with a large number of DNA lesions, which are generated by intrinsic (e.g., reactive oxygen species) and extrinsic sources (e.g., by ionizing radiation, ultraviolet radiation). In addition to DNA damage-induced lesions, the cells have to deal with spontaneous lesions, such as AP (apurinic/apyrimidinic) sites or the deamination of bases [8]. If left unrepaired or repaired incorrectly, DNA damage may lead to cell death, genomic instability, and mutagenesis. To keep genome stable and secure cellular homeostasis, it is essential for the cells to counteract DNA damage by activating the DNA damage response (DDR), which finally coordinates cell fate decision making [9]. The activation of DNA repair (orchestrated by set of DNA damage response proteins, including BRCA1, NBS1, as well as repair proteins Ku70/80 and others) and cell-cycle checkpoints (regulated by MDC1, 53BP1 and checkpoint kinases Chk1/Chk2) act as an immediate response to DNA damage to provide protection and recovery of injured cells, whereas activation of cell death occurs much later and aims to eliminate the irreversibly damaged cell. Depending on the type and the severity of stimulus and cellular context, DNA damage can induce cell-cycle arrest, senescence or different cell death programs, such as mitotic catastrophe, apoptosis, autophagy and necrosis [10,11]. Activation of p53 was reported to be crucial for the initiation and progression of senescence and apoptosis following DNA damage in most cell types [10]. Activated p53 further regulates a series of its apoptotic target genes, such as cyclin dependent kinase inhibitor 1A (p21), p53-upregulated modulator of apoptosis (PUMA), p53AIP1, BCL2 associated X (BAX), as well as several kinds of miRNAs including miR-34a, leading to cell apoptosis [10]. Many other kinds of regulators and molecules including Caspase-2, Bcl-2, Nur77, TSC2/mTORC1 signaling pathway and JNK signaling pathways are involved in the regulation of cell death following DNA damage [10,12]. DNA double-strand breaks (DSBs) are the most cytotoxic DNA lesions, which may cause disruption of chromatin structure, including chromosomal deletions, insertions, duplications, and translocations, which further cause cell death [13].

A series of DNA damage repair pathways has evolved in cells to repair different types of damage, including homologous recombination repair (HRR), nonhomologous end-joining (NHEJ), base excision repair (BER), nucleotide excision repair (NER), and mismatch repair (MMR) [14]. HRR and NHEJ are responsible for DNA double-stranded breaks (DSBs) which are caused by ionizing radiation, DNA replication stress or chemotherapeutic agents. BER is mainly responsible for the repair of DNA single-stranded breaks (SSBs), which are caused by alkylating agent or reactive oxygen species. NER plays a key role of repairing SSBs that are caused by ultraviolet light or chemotherapeutic drugs such as cisplatin. MMR corrects the DNA double helix mismatch of base pairs. Genomic instability caused by defects in the DDR is an important basis of cancer initiation and progression [15], therefore, targeting the DDR pathway is a very feasible strategy for cancer treatment.

Small-molecule inhibitors focused on the DDR pathway can target abnormally expressed proteins in cancer cells and have yielded promising therapeutic effects. Several small-molecule inhibitors targeting DNA damage check points (ATM inhibitors [16,17], ATR inhibitors [18], CHEK1/2 inhibitors [19,20], and poly (adenosine diphosphate-ribose) polymerase inhibitors [21] (PARPis) or DNA repair pathway proteins (RAD51 inhibitors [22,23], and FEN1 inhibitors [24]) have been designed and generated under preclinical tests or clinical trials [25]. PARPis have garnered worldwide attention for their excellent curative effect. Several PARPis have been clinically used for treatment of several kinds of cancers, including breast cancer and ovarian cancer. In 2005, two groups described the synthetic lethality (SL) interaction between PARP inhibition and *BRCA1* or *BRCA2* mutation, suggesting a novel strategy for treating patients with *BRCA*-mutant tumors [26,27]. SL is a concept introduced nearly a century ago by geneticists to describe the situation whereby a defect in either one of two genes has little effect on the cell or organism but a combination of defects in both genes results in death [28]. In December 2014, olaparib was approved in the European Union and United States for the treatment of advanced ovarian cancer with *BRCA* mutations, this marked the first clinical acceptance of the feasibility of PARP1 as an anti-tumor target and the SL theory. Another PARPi, rucaparib, received accelerated approval in the United States in December 2016 for advanced ovarian cancer with *BRCA* gene mutations and two or more chemotherapy treatments. In March 2017, niraparib was approved by the FDA for maintenance therapy in patients with recurrent epithelial ovarian, fallopian tube, and primary peritoneal cancer. Based on the successful application of PARPi in *BRCA*-deficient breast cancer and ovarian cancer, PARPis have become a new research focus, with many clinical trials for the treatment of metastatic PC (mPC) showing good efficacy by the mechanism of synthetic lethality [26,27]. However, these inhibitors are also associated with problems such as drug resistance. Thus, it is necessary to explore the mechanisms of drug resistance and possible combination treatments that can enhance drug sensitivity. This paper will specifically discuss: (1) the mechanism of action of PARPis; (2) the application of PARPis in the precision treatment of castration-resistant prostate cancer; (3) the current progress of clinical research; (4) the mechanism of PARPis’ drug resistance; (5) strategies for combination treatments and aims to provide insights into the clinical treatment of PC.

## 2. PARP Proteins

PARPs are nuclear enzymes that catalyze poly ADP ribosylation in most eukaryotic cells. They are involved in the posttranslational modification of various proteins. The PARP family includes 17 subtypes, including PARP1, PARP2, PARP3, and PARP7. PARP1 is the earliest and most intensively studied subtype of the PARP family. It is a rich ribozyme encoded by ADPRT1 (ADP ribozyme transferase-1 gene) at q41-42 on chromosome 1 and has an N-terminal zinc finger domain, namely, a DNA binding domain (DBD), a central self-modifying domain (AD), and a C-terminal catalytic domain (CAT) [27]. It participates in various functions, including DNA methylation; cell apoptosis; cell proliferation and differentiation; gene transcription regulation; chromatin remodeling; and, most importantly, DDR [29]. PARP1 is a DNA damage sensor and signal transducer that can recognize a wide range of damaged DNA structures including DSBs, single-strand breaks, gaps, and a range of non–B-form structures in which the base-stacking continuity of at least one strand is disrupted [30], and activates the catalytic process of PARP1. PARP1 catalyzes the decomposition of NAD+ into nicotinamide and ADP ribose, then takes ADP ribose as a substrate to make the receptor protein that includes PARP itself, together with histones and other chromatin-associated proteins, forming the poly (ADP-ribose) (PAR) chains [4,31]. This PARylation of proteins in the vicinity of the DNA breaks then likely mediates DNA repair by modifying chromatin structure (e.g., via histone-PARylation) and by localizing DNA repair effectors (e.g., XRCC1). PARP1 autoPARylation eventually leads to its own release from the site of DNA damage [32,33].

## 3. The Mechanism of Action of PARP Inhibitors

**Figure 1:**
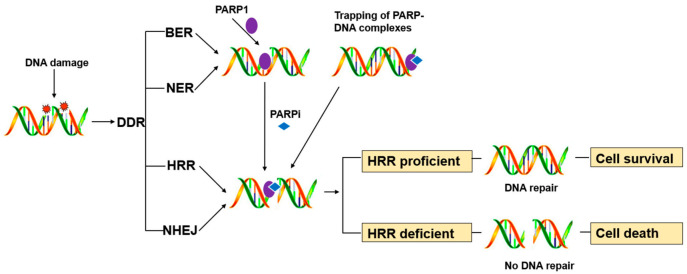
Schematic of synthetic lethal mechanism.

PARP1 is a key enzyme in SSB repair (SSBR), and the PARP inhibitors were initially developed as catalytic inhibitors to block SSB repair [33]. PARP1 has been strongly implicated in promoting SSBR [34]. Direct SSBs are detected by PARP1, which ribosylates itself and, most likely, other proteins in the vicinity of the break (e.g., histones). PARP1 activity results in recruitment/retention of the putative chromatin remodeling proteins APLF and ALC1, and importantly also XRCC1, a scaffold protein that promotes retention and/or activity of core SSBR enzymes including factors required to process damaged termini [35]. In cells, PARP1 inhibition causes failure of SSB repair. Furthermore, persistent SSBs have been shown to stall and collapse the replication fork, leading to DSB [35,36].HR-competent cells can repair DSBs for cell survival, whereas in cells with defects caused by mutations in breast cancer-associated antigens (BRCA1 or BRCA2) or other HR-associated proteins, the damage remains unrepaired, leading to cell death (Figure 1) [13].

PARP inhibitors have also been reported to trap PARP enzymes in DNA. PARP inhibitors captured the PARP1 and PARP2 enzymes at the DNA damage sites, trapped PARP-DNA complexes were more cytotoxic than unrepaired SSBs caused by PARP inactivation, implying that PARP inhibitors act, in part, as toxins that trap PARP enzymes on DNA. The toxic PARP-DNA complex prevents replication fork progression and leads to cell death unless the damage is repaired. In addition, among the inhibitors, niraparib-treated cells were more capable of trapping than olaparib or veliparib-treated cells [37,38].

A third mechanism of PARP inhibitor sensitivity was discovered recently [39,40]. DSBs are dominantly repaired by HR in the S and G2 phase when sister chromatids are present [41]. In the presence of HR defects, tumor cells must rely on another microhomology-mediated end-joining or Alt-EJ pathway for repair. The pathway is dependent on PARP1 and translational ion polymerase (POLQ) [42]. Indeed, PARP1 is required to effectively recruit POLQ to DSBs. Therefore, inhibitors of PARP1 or POLQ can block the Alt-EJ pathway and kill HR-deficient tumor cells.

## 4. DNA Damage Repair Gene Defects in Prostate Cancer

Genomic instability is a hallmark of cancer and often results from changes in the ability of tumor cells to repair DNA. DDR gene defects are common in different types of cancer, such as breast cancer and ovarian cancer [43,44,45]. DDR gene mutations are also common in PC, especially in advanced PC [46]. Multiple studies have reported an association between frequent germline and somatic deleterious mutations in DDR genes and advanced PC, paving the way for the use of PARPis in the treatment of PC [47]. The Cancer Genome Atlas (TCGA) contains 333 comprehensive exome sequences from patients with primary PC. Tumors with a Gleason score of ≥8 were present in 26% of the entire cohort, and 62 of the 333 patients (19%) had harmful germline or somatic mutations in key genes in the DDR pathways (*BRCA2*, *BRCA1*, *CDK12*, *ATM*, *FANCD2*, *RAD51C*) 5. Colin et al. isolated germline DNA from 692 samples from male patients with metastatic PC without a family history of cancer and used multisequencing analysis to evaluate mutations in 20 DNA repair genes associated with chromosomal-dominant cancer susceptibility syndrome 5. The analysis showed that 11.8% of patients with mPC and 4.6% of patients with localized PC carried germline deleterious mutations in DDR genes. These mutations were found in the *BRCA2* (37 patients (5.3%)), *ATM* (11 patients (1.6%)), *CHEK2*(10 patients (1.9%)), *BRCA1* (6 patients (0.9%)), *RAD51D* (3 patients (0.4%)) and *PALB2* (3 patients (0.4%)) genes. Mateo et al. conducted a phase II clinical trial in which 16 (33%) of 49 metastatic castration-resistant prostate cancer (mCRPCs) were identified as pure and absent or deleterious mutations (or both) in DDR genes (including *BRCA1/2*, *ATM*, *CHEK2*, and the genes for Fanconi anemia) [7]. The dream team of Stand Up 2 Cancer and the Prostate Cancer Foundation (SU2C-PCF) [48] identified AR mutations or amplifications (62.7%), TP53 mutations or deletions (53.3%), PTEN deletions (40.7%), RB1 deletions (8.6%), BRCA1/BRCA2 mutations or deletions (14.6%), and CDK12 mutations (4.7%) in metastatic biopsy samples from 150 patients with mCRPC. Recently, Dall et al. analyzed the change, distribution, and phenotype of 24 genes related to DNA repair in 944 cases of prostate cancer, including the primary PC tumor and metastasis sites [49]. Their results showed that defects occurred in DDR genes in 20% of primary PC tumors, occurring most commonly in *BRCA* (11.4%) and *ATM* (5.8%). Although they did not distinguish between germline and somatic mutations, they found a higher mutation rate (nearly 35%) in metastatic lesions.

In summary, DDR gene mutations occur in all types of PC, whether it is localized, metastatic, or castrated. We have summarized some of the trial data and presented it in Table 1. Note that the mutation frequencies of the listed genes vary heavily across the different studies. This variation may be due to the different trials with different PC types or racial differences in patients. We also found that the most-commonly mutated DDR gene is *BRCA2*, which is present in 8–12% of mPCs; conversely, *BRCA1* mutations are rare, occurring in approximately 1% of mPCs. ATM mutations were the second most common, accounting for approximately 6–8%. Other commonly mutated or missing homologous recombination (HR) genes in mPCs include *FANCA*, *CHEK1*, *CHEK2*, *PALB2*, and *RAD51*; although the frequency of each gene mutation is low, these unusual mutations occur in 5–10% of mPC [49].

## 5. Current Progress in Clinical Research on PARPis for the Treatment of PC

PARPis have shown good efficacy in tumor therapy. Olaparib has been applied successfully to advanced ovarian and breast cancers with *BRCA* gene mutations [55]. For example, the first successful use of PARPis in the treatment of advanced breast cancer in the OlympiAD phase III trial showed that PARPis offered significant benefits over standard chemotherapeutic agents [56]. In total, 84 patients with advanced breast cancer with BRCA1 or BRCA2 mutations were enrolled in the ABRAZO II clinical trial, which was divided into two cohorts: Cohort 1 contained patients who had responded to prior platinum therapy; Cohort 2 contained patients who had received at least second-line chemotherapy but had not been treated with platinum. The results showed that the objective response rate (ORR) of the two cohorts was 21% and 37%, with median progression-free survival (PFS) of 4.0 months and 5.6 months, respectively, with talazoparib monotherapy, suggesting that talazoparib monotherapy can be used for the treatment of advanced breast cancer after previous chemotherapy, with better efficacy and good safety [57].

In addition, most published clinical data support the use of PARP inhibitors in patients with ovarian cancer and *BRCA1/2* mutations. Three phase III clinical trials (NOVA, SOLO2, and ARIEL3 [58,59,60]) showed significant improvements in PFS and led to the approval of three PARP inhibitors-olaparib, niraparib, and rucaparib-as maintenance treatments for platinum-sensitive, recurrent ovarian cancer.

The successful use of PARPis in *BRCA*-deficient advanced breast and ovarian cancer has led to further investigation of the efficacy of PARPis in PC. Therefore, it is relevant to discuss the current status of clinical research into PARPis for the treatment of PC.

### 5.1. Olaparib

The TOPARP-A phase II clinical trial conducted by Fong et al. provided olaparib therapy to 49 patients with mCRPC with germline and somatic DDR gene mutations [55]. In this study, olaparib had a response rate of 32% over the whole cohort (defined as PSA reduction, objective tumor remission, or CTC reduction). However, when patients were stratified based on NGS results, 14 of 16 patients with HRR mutations responded well to olaparib. In contrast, only two of 33 patients with NGS without HRR mutations responded well to olaparib. This study revealed that olaparib was effective in patients with DDR mutations.

Subsequently, Mateo et al. conducted a phase II TOPARP-B clinical trial to verify the association between DDR gene abnormalities in mCRPC and olaparib response [61]. Patients received 400 mg or 300 mg olaparib twice daily, and the primary end point was defined in all patients as any one of the following results: a radiographic objective response (according to a Response Evaluation Criteria in Solid Tumors version 1.1 evaluation), a decrease in prostate specific antigen (PSA) of 50% or more compared with the baseline or conversion (from the baseline circulating blood tumor cell count measured every July of ≥5 cells/mL to <5 cells/mL). This clinical study enrolled patients with mCRPC, who were 18 years of age or older, and had previously received one or two taxanes. A 54% overall composite response was observed in the 400 mg dose cohort and a 39% response was observed at the 300 mg dose level. The overall response rate for men with *BRCA1/2* changes was 83%, and 20% for men with no other specific changes. These studies showed that olaparib appears to be primarily effective in mCRPC patients with homologous recombination deficiency (HRD) changes driven by BRCA1/2 mutations. TOPARP-A and TOPARP-B studies have shown that tumors with *BRCA1* or *BRCA2* alterations were more sensitive to olaparib monotherapy than tumors with alterations in any other gene associated with HRR.

The recent phase II trial, PROfoundIII, conducted by Bono et al., assessed the clinical efficacy of olaparib in mCRPC patients treated with enzalutamide or abiraterone [52]. These patients had previously received either at least one of the second-generation hormone drugs or a type of chemotherapy (taxanes) and had HRD genetic changes. The patients were divided into two cohorts: Cohort A comprised patients with *BRCA1*, *BRCA2*, and *ATM* mutations; Cohort B comprised patients with mutations in one of the other 12 HRD genes (*BRIP1*, *BARD1*, *CDK12*, *CHEK1*, *CHEK2*, *FANCL*, *PALB2*, *PPP2R2A*, *RAD51B*, *RAD51C*, *RAD51D*, or *RAD54L*). The primary endpoint of this study was radiographic progression-free survival (rPFS). In total, 387 patients who met the criteria were randomly assigned to treatment. In Cohort A, the median PFS and the median duration of pain progression were significantly longer in the olaparib group than in the control group. The median overall survival was 18.5 months in the olaparib group and 15.1 months in the control group (the risk ratio for death was 0.64; 95% CI, 0.43–0.97; *p* = 0.02). In the independently reviewed control group, 81% of patients showed radiographic evidence of disease progression. Of the assessable patients, 43% (66 out of 153) in the olaparib group and 8% (6 out of 77) in the control group had a prostate-specific antigen (PSA) decline of 50% from the baseline (PSA50) response. Circulating tumor cell clearance was 30% (29 out of 97) in the olaparib group and 11% (5 out of 44) in the control group. These data indicated that the study met the primary endpoint for rPFS. The study further demonstrated the significant efficacy of olaparib in patients with mCRPC with germline or HRD mutations. In January 2020, the positive results of the PROfound trial prompted the FDA to approve olaparib for the treatment of mCRPC in patients with germline or somatic HRR gene mutations, followed by new hormone therapy (NHA).

### 5.2. Rucaparib

In the TRITON2 phase II trial, 52 patients with deleterious germline or somatic alterations in BRCA1, BRCA2, or one of 13 other prespecified HRD genes received 600 mg rucaparib twice daily [62]. Patients with mCRPC receiving one or two androgen-receptor-guided therapies and one taxonomic chemotherapy were eligible. The primary endpoint of ORR was identified in a centralized assessment of patients with measurable disease based on RECIST V1.1 evaluation, and PSA responses (50% reduction) were identified in patients with no measurable disease. When the initial data were reported at ESMO 2018, 11 of 23 patients with *BRCA* mutations were confirmed to have prostate-specific antigen responses (47.8%). In addition, among the 11 *BRCA* patients, five (45.5%) patients were confirmed to have responses via imaging, and only one (0.09%) had to discontinue rucaparib therapy due to toxicity. Based on these data, the FDA awarded rucaparib a breakthrough treatment title in late 2018 for patients with mCRPC with *BRCA 1/BRCA2* mutations. The latest results of this trial were presented at the 2019 Annual Meeting of the American Society of Clinical Oncology (ASCO): 136 patients received rucaparib, of whom 62 had *BRCA2* mutations, seven had *BRCA1* mutations, 41 had *ATM* changes, 14 had *CDK12* changes, and 12 had other changes in DDR [63]. Among the patients with *BRCA1/2* mutations in germline or somatic cells, 44.0% (11/25) had a definite imaging response and 51.1% (23/45) had a definite PSA response. Based on good preliminary results from TRITON2, the FDA approved rucaparib for androgen-receptor-guided therapy and paclitaxel-based chemotherapy in patients with mCRPC with harmful *BRCA* mutations (both germline and somatic). Besides, ORR and PSA responses were also reported in patients with non-*BRCA* gene alterations, as follows: *ATM* (10.5% vs. 4.1%), *CDK12* (0% vs. 6.7%), *CHEK2* (11.1% vs. 16.7%). Importantly, patients with *PALB2*, *FANCA*, *BRIP1,* and *RAD51B* mutations presented a response contrary to patients with germline or biallelic loss of *ATM*. In contrast to *BRCA*-mutated tumors, the ORR and PSA responses in patients with *ATM*, *CHEK2* and *CDK12* mutations were low. On the one hand, the TRITON2 study confirmed safety and efficacy in *BRCA*-mutated tumors, on the other hand, it revealed no accurate biomarkers in non-*BRCA*-mutated tumors, warranting further investigation [64,65].

### 5.3. Niraparib

The GALAHAD phase II study tested 39 patients with mCRPC with *BRCA1*, *BRCA2*, *ATM*, *FANCA*, *PALB2*, *CHEK2*, *BRIP1*, or *HDAC2* mutations [66]. The patient population was very similar to that of TRITON2. The preliminary results were reported at the ASCO Urogenital Cancer Symposium in early 2019. In 23 patients with *BRCA* mutations, 38% (5/13) ORR of RECIST, 57% (13/23) PSA response (50% reduction), and 48% (11/23) CTC conversion from >5 to <5 was observed. In the 16 patients without *BRCA* mutations, ORR, PSA, and CTC conversion rates were 11% (1/9), 6% (1/16), and 31% (5/16), respectively.

### 5.4. Talazoparib

Similar to TRITON2, TALAPRO-1 was a phase II study to test talazoparib in a post-taxane mCRPC population [67]. The primary end point of this study was ORR. Preliminary data from the phase II PARPis trial indicated a PSA response rate of more than 50% and a soft tissue response rate of approximately 40% in patients with *BRCA1/2*-mutated mCRPC who had undergone at least a range of taxane-based chemotherapy or AR signaling inhibitors, such as abiraterone and enzalutamide. PARPi activity in PC with non-BRCA mutations remains to be confirmed. In general, PARPi was well tolerated in the mCRPC population after chemotherapy.

These clinical trials show that the good efficacy of PARPis was achieved in *BRCA1/2*-mutated mCRPC. Based on its good curative effect, olaparib has been approved for the treatment of PC with *BRCA* mutations by FDA. Rucaparib has been approved for use in combination with androgen receptors to guide therapy and chemotherapy based on paclitaxel harmful *BRCA* mutations (species and somatic cell) in patients with mCRPC. Consequently, this will support the clinical trials of other PARPis, such as veliparib and talazoparib. Key clinical trial data for these four PARP inhibitors in PC patients are presented in Table 2.

### 5.5. PARPi Toxicity and Its Limitations

Compared to chemotherapy, the toxicity of PARP inhibitors is low; however, there is a lack of mature data about the long-term safety of PARPi in PC populations. According to the TOPARP-AII trial, the most common ADR event was anemia (20%) and fatigue (12%) [55]. In the TOPARP-BII trial [61], the adverse event was anemia (31% in the 300 mg cohort and 37% in the 400 mg cohort); in the PROFOUND trial, the most common adverse events were hematological (anemia 46%), gastrointestinal (nausea 41%; loss of appetite 30%), and fatigue or asthenia (41%). Twenty-two percent of the patients required dose reduction due to adverse events [62]. In the PROFOUND trial, myelodysplastic syndrome was not reported, despite initial reports and published case reports [69]. A recently published meta-analysis of 14 trials showed that PARPi in combined therapy may be associated with myelodysplastic syndrome, but the incidence of that complication is low [70].

## 6. PARPi Drug Resistance and Combination Strategies

### 6.1. Clinical Development of PARPi Resistance in PC

**Figure 2:**
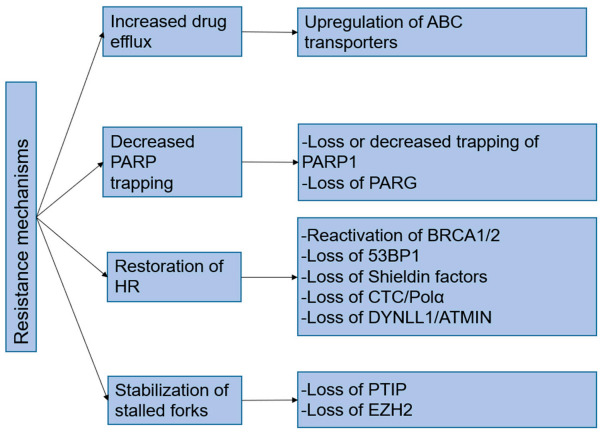
Mechanism of resistance.

Although PARPi can cause a response in patients with *BRCA* mutations, some patients may develop resistance [71]. As most patients with mCRPC with harmful *BRCA* mutations (germline and somatic) are now receiving the FDA-approved rucaparib for androgen-receptor-guided therapy and paclitaxel-based chemotherapy, the number of patients receiving treatment will increase in the next few years, enabling large-scale studies of the mechanisms of drug resistance. Extensive in vitro and in vivo studies have identified several mechanisms of drug resistance: (i) effects on PARPi cell utilization; (ii) direct effects on PAR chain activity and abundance; (iii) PARPis leading to HR reactivation; (iv) restoring the stability of the replication fork [68]. These mechanisms are summarized in Figure 2.

In ovarian or breast cancer, olaparib resistance is associated with HRR recovery, including the reversal of *BRCA2* mutations. Whether the same mechanism applies to PC and whether it can be detected in liquid biopsy remains under investigation [13]. Some clinical trial data have provided relevant evidence: One patient with mCRPC with a germline *BRCA2* mutation was treated with sequential carboplatin and rucaparib. Genomic analysis of the available baseline tumor and blood samples over time using next-generation sequencing panels revealed polyclonal reversal of *BRCA2* mutations following carboplatin treatment but prior to rucaparib treatment. In, total 12 cell reversal mutations were detected, ranging from small insertions deletions to large insertions of up to 387 amino acids. These changes were all predicted to restore the open reading frame and potential protein function of *BRCA2*, most likely because these reversal mutations were observed before treatment. David et al. studied a patient with germline *BRCA2* mutations [71]. The patient was initially diagnosed with localized PC (Gleason score of 6) and was treated with radiation in 2009. Despite a series of androgen-directed therapies, the disease later recurred and progressed. The patient was treated with the PARPi talazoparib in 2014 and initially responded well; at 1 month after treatment, his PSA level dropped from an initial 16.52 to the lowest observed level of 0.66. However, imaging and PSA tests showed the progression of the disease several months later. This study was the first to identify a reversal of a *BRCA2* mutation associated with drug resistance to olaparib and talazoparib in PC.

There is emerging evidence that DNA replication fork protection also contributes to PARPi resistance. For example, Rondinelli et al. found that low EZH2 levels reduced H3K27 methylation, prevented MUS81 supplementation in the stasis fork, and led to replication fork stabilization. Therefore, in *BRCA2*-deficient cells, loss of EZH2/MUS81 axis function promoted PARPi resistance. Low EZH2 or MUS81 expression levels predict chemical resistance and a poor prognosis in patients with *BRCA2* mutated tumors [72].

DNA terminal excision is the key to the selection of different DNA repair pathways, and DNA terminal excision may determine different repair outcomes and PARPi sensitivity [73]. Several recent reports have suggested that DNA terminal excision is involved in PARPi resistance. DNA terminal excision is dependent on cell-cycle-dependent kinase (CDK) activity. A study showed that CDK12 deficiency reversed both primary and secondary PARPi resistance in triple-negative breast cancer (TNBC) in both BRCA wild-type and mutant type [74]. In addition to CDK, cofactors such as 53BP1, Rev7 and RIF1 also play a significant role in DNA terminal excision and PARPi resistance [21,75,76].

Epigenetic modifications may also influence PARPi resistance [13]. Fukumoto et al. found that m6A promoted PARPi resistance in *BRCA*-deficient epithelial ovarian cancer cells by stabilizing FZD10 and upregulating the Wnt/β-catenin pathway [21]. Pharmacological changes also modulate the response to PARPi inhibitors. PARPi is the substrate of a multidrug-resistant protein encoded by the ABCB1 gene [77]. In vivo and in vitro studies have shown that an enhanced multidrug-resistant protein-mediated drug efflux contributes to the acquired resistance of PARPis [78,79].

### 6.2. Combination Strategies for PARPis

When used alone, PARPis have shown good antitumor activity in HR-deficient cells, especially in cells with *BRCA1/2* mutations. However, drug resistance still limits the population of patients that will benefit from treatment. To expand this population, overcome emerging resistance; and improve the depth, duration, and overall survival of the response, it is necessary to study the use of PARPis in combination with other therapeutic agents [47,80]. Although the first foray into the clinic for PARP inhibitors was in combination with DNA-damaging chemotherapy, clinical development was limited by the more-than-additive toxicity, in particular dose-limiting myelosuppression. PARPis have also been studied in combination with several other reagents, such as USP7 inhibitors, androgen deprivation therapy, and immunotherapy. Francesco et al. tested the efficacy of USP7 inhibitors combined with PARPis as a novel treatment option for advanced PC [81]. This study investigated whether the pharmacological inhibition of USP7 could impair the AR-dependent proliferation of PC cells by damaging the stability of AR. The results showed that the pharmacological inhibition of USP7 could lead to downregulation of the CCDC6 protein and DNA repair of gene defects, making CRPC cells sensitive to PARPis, either alone or in combination with standard radiotherapy.

Clarke et al. conducted a phase II clinical trial that enrolled 171 patients with mCRPC who had not received more than two chemotherapy treatments or second-generation antihormone drugs [82]. Patients were randomly assigned (in a 1:1 ratio) to both abiraterone and olaparib, with the primary end point being radiographic progress-free survival (rPFS) as assessed by the investigators. The median rPFS for olaparib and abiraterone was 13.8 months (95% CI 10.8–20.4), compared with 2 months (5.5–9.7) for placebo and abiraterone (hazard ratio (HR) 0.65, 95% CI 0.44–0.97, *p* = 0.034). From the data, it was concluded that olaparib combined with abiraterone can provide greater clinical efficacy in the treatment of mCRPC than abiraterone alone. These data suggest that the combination of olaparib and abiraterone may provide additional clinical benefits for a wide range of mCRPCs. Moreover, a randomized, open-label phase III trial, conducted by Bono et al., assessed the effect of olaparib in men with mCRPC who were undergoing treatment with new hormone drugs, such as enzalutamide or abiraterone. The primary endpoint of this study was rPFS.

In addition, extensive research is assessing whether PARPis have greater efficacy when combined with immune checkpoint inhibitors. Unlike PARP inhibition, which plays a role in promoting cancer cell death, new evidence suggests that PARP inhibition can enhance the response of immune checkpoint inhibitors. According to the immune status, immune checkpoint inhibitors (ICI) such as anti-PD-1/PD-L1 and anti-CTLA-4 are effective anti-tumor agents and have been approved in a variety of cancers. PARPi-mediated unrepaired DNA damage regulates the tumor immune microenvironment through a series of molecular and cellular mechanisms, such as increased genomic instability, immune pathway activation and upregulation of PD-L1 expression in cancer cells-this may enhance the response to ICIs. In this case, PARPis and ICIs yield an excellent combination effect [83]. In studies by Jiao et al., PARPis upregulated PD-L1 expression in breast cancer cell lines and animal models by inactivating GSK3. PARPis weakens anticancer immunity by upregulation of PD-L1 and blocking PD-L1, resensitizing PARP-treated cancer cells to T cells. They confirmed the crosstalk between PARP inhibition and the PD-L1/PD-1 immune checkpoint axis and concluded that in vivo therapeutic efficacy was significantly improved for the combination of PARPis and anti-PD-L1 therapy compared with the efficacy of each drug alone [84]. In addition, BMN67 (a PARPi) showed immunomodulatory effects in BRCA-deficient mouse models of ovarian cancer [85]. In mCRPC, the combination of olaparib and durvalumab resulted in a PSA response (a reduction of ≥50%) in 47% (8 of 17) patients. Patients with DDR gene abnormalities experienced greater benefits than those who did not respond, suggesting that DDR gene abnormalities lack a biomarker that can predict response. These encouraging preclinical and clinical studies have shown that the combination of PARPis and ICIs offer significantly improved efficacy compared with PARPis alone.

In primary and acquired HR-proficient cancers, strategies to augment the response to PARPis will represent a major advance in cancer treatment. The study of Karakashev et al. showed that BET bromodomain inhibition and PARP inhibition show synergistic effects in BRCA-related ovarian cancers caused by mitotic line inhibition. The G2-M cell cycle checkpoint adjustment factor, WEE1, and the DNA damage response factor, TOPBP1, may be downregulated in BRCA-deficient ovarian cancer following treatment with BET inhibitor JQ1. When BET inhibitors (BETis) are combined with PARP inhibitors, DNA damage checkpoint defects and collaborative increases were observed, consequently, cells with an accumulation of DNA damage could still enter mitosis, eventually leading to mutations [86]. Studies by Fehling et al. showed that JQ1 reduced C-MYC and increased DNA damage and apoptosis, and that the BETi JQ1 sensitized cholangiocarcinoma (CCA) tumor cells to PARPis. Their data suggested that the combination of BETis and PARPis may be a promising strategy for the treatment of CCA [87]. In the study of Lu Yang [88], three BETis (JQ1, I-BET762, and OTX015) showed strong synergistic effects with olaparib in breast cancer (MDA-MB-231), ovarian cancer (OVCAR10), and prostate cancer (VCaP), for which PARPi treatment has been proposed in clinical trials.

## 7. Conclusions and Future Perspectives

The successful development of PARPis over the past 10 years has provided an effective treatment option for some ovarian and breast cancers, and the clinical application of PARPis inPC, especially in mCRPC, has brought new hope to patients. Examples include olaparib, which has been approved by the FDA for PC with BRCA mutations; rucaparib, which has been approved for androgen-receptor-guided therapy and paclitaxel-based chemotherapy for patients with mCRPC and harmful *BRCA* mutations (germline and somatic); and two other PARP inhibitors, veliparib and talazoparib, which are under investigation in clinical trials. However, the emergence of drug resistance to PARPis in patients may limit their clinical application; thus, revealing the mechanism of drug resistance to PARPis in patients with PC may bring new hope and direction for strategies related to the combination of PARPis and other drugs (such as hormone drugs). A large amount of data have emerged in the past decade, allowing assessment of the potential of PARPis combinations that have shown improved efficacy in combination with USP7, second-generation antihormone drugs, immunotherapy, and BETis. However, a major challenge faced is how to reclassify PARPis and administer them with the appropriate combination drug.

Simultaneously, more preclinical studies and clinical trials are needed to better understand how changes other than those in *BRCA1/2* confer sensitivity to PARPis to different types of tumors. Intrapatient tumor heterogeneity, genomic tumor evolution, and standardization of tumor and ctDNA NGS assays need to be better addressed to facilitate the incorporation of genomic stratification of PC in patient care. Molecular profiling beyond mutation calling may offer certain advantages for stratifying patients. Non-gene-specific biomarkers currently in clinical development include mutational patterns or signatures associated with HR defects [89], genome-wide evaluation of copy number and loss of heterozygosity (LOH) changes [90], and protein-based markers of HR proficiency [91].

## Figures and Tables

**Table 1:** Frequency of genetic mutations in different types of prostate cancer.

					Classification of Prostate Cancer								
					mPC		Located PC		mCRPC				
DDR Classification			Different Studies	A Prospective Pilot Study Using a 37 Gene Panel [50]	Several Academic Centers in the UK and USA [7]	Tcga [5]	Several Academic Centers in the Uk and Us [7]	Su2c-Pcf Dream Team [48,51]	Toparp-a [6]	Toparp-Bii [52]	Whole Exome Sequencing [53]	Sequencing of Dna Repair Genes [54]
		FOGM ^1^										
	DDR Genes Mutations											
*HR*	*BRCA1*				0.87%	1%	0.6%	0.7%	6%	/		0.3%
	*BRCA2*			6.25%	5.35%	3%	0.2%	13.3%	14%	7%	2.9%	5%
	*CDK12*					2%		4.7%	/	6%	3.3%	0.6%
	*ATM*			4.7%	1.59%	4%	1%	7.3%	10%	40%	3.8%	0.3%
	*CHEK2*			3.1%	1.87%	0%	0.4%	3%	/	/		
	*TP53*			7.8%		0%		/	/	/		0.3%
	*PALB2*			4.7%	0.43%	0%	0.4%	2%	/	3.5%	1%	0.6%
	*XRCC2*			1.6%	0%			/	/	/		
	*RAD51D*				0.4%		0.2%					
	*RAD51C*				0.14%	3%	0.4%					
MMR	*MLH1*				0	0.3%		0.7%				
	*MSH2*			1.6%	0.14%	0.3%	0.2%	2%				
	*MSH6*			1.6%	0.14%	1.5%	0.2%	1%				
NHEJ	*PRKDC*							8%				
NER	*ERCC2*			15.4%	0.6%	0.6%		1.3%				0.3%
	*ERCC5*				0.3%	0.3%		1.3%				

^1^ FOGM-Frequency of genetic mutations.

**Table 2:** Summary of progress in clinical trials of PARP inhibitors for mCRPC.

Trial ID	PARPis	Clinical Trials	Patient Population	PSA Response Rate	Adverse Events	Conclusions
NCT01682772	Olaparib	TOPARP-A-II [6]	mCRPC patients who received abiraterone or enzalutamide, and cabazitaxel before.	16 of 49 (33%) patients, (95%, 20–48)	Anemia (20%) and fatigue (in 12%)	Olaparib has been shown to be effective in PC patients who were no longer responding to standard treatments and who had defects in DNA repairs genes
NCT01682772	Olaparib	TOPARP-B-II [52]	mCRPC patients: (1) previously treated with one or two taxane chemotherapy regimens; (2) esisting DDR gene mutations.	100% of BRCA2 and FANCA mutated mCRPC drop ≥50% baseline	Anemia (31% in the 300 mg cohort and 37% in the 400 mg cohort)	Olaparib has antitumor activity against mCRPC with DDR gene aberrations
NCT02987543	Olaparib	PROfound-III [62]	mCRPC pts who had disease progression while receiving enzalutamide or abiraterone and had at least one HRR gene mutation.	30% (73 of 243) in the olaparib group and 10% (12 of 123) in the control group	Anemia and nausea	Rucaparib had better anti-tumor efficacy than enzalutamide or abiraterone in mCRPC pts with at least one gene mutation.
NCT02952534	Rucaparib	TRITON2-II [63]	mCRPC pts with a deleterious germline or somatic alteration in *BRCA1*, *BRCA2* or 1 of 13 other prespecified HRR genes.	11 of 23 (47.8%) of *BRCA*-mutated; (95% CI, 26.8–69.4)	Nausea (48.1%) and asthenia/fatigue (44.2%)	Rucaparib has encouraging antitumor activity in mCRPC pts with a deleterious alteration in *BRCA1* or *BRCA2*.
NCT02854436	Niraparib	GALAHAD [67]	mCRPC pts with DRD and with disease progression on taxane and androgen receptor-targeted therapy.	57% (95% CI, 34–77)	hematologic anemia (29%), thrombocytopenia (15%) and neutropenia (7%)	Niraparib demonstrates clinical activity in pts with treat-ment-refractory mCRPC with durable responses particularly in biallelic *BRCA* mutation carriers.
NCT03148795	Talzoparib	TALAPRO-1 [68]	DDR-mutated mCRPC progressed on a taxane or androgen-receptor signaling inhibitor	Not completed	Not completed	Not completed

## Data Availability

Not applicable.

## Abbreviations

PARP	Poly (adenosine diphosphate-ribose) polymerase	NHEJ	Nonhomologous end joining
PARPis	Poly (adenosine diphosphate-ribose) polymerase inhibitors	DSB	Double-stranded break
HRR	Homologous Recombination Repair	C-NHEJ	Classical non-homologous terminal connections
PC	Prostate Cancer	Alt-EJ	Alternative terminal connections
FDA	Food and Drug Administration	SSA	Single-strand annealing
mCRPC	Metastatic castration resistant prostate cancer	SSB	Single strand break
ADT	Androgen deprivation therapy	PSA	Prostate-specific antigen
AR	Androgen receptor	CTC	circulating tumor cell
CRPC	Castration-resistant prostate cancer	NGS	Next-generation sequencing
DDR	DNA damage repair	rPFS	radiographic progression-free survival
USP7	Ubiquitin-specific protease 7	NHA	New hormone therapy
BET	Bromodomain and extra terminal domain	ORR	Objective response rate
ATM	Ataxia telangiectasia mutated	EMSO	European Society for Medical Oncology
ATR	Ataxia- and Rad-related	ASCO	American Society of Clinical Oncology
CHEK1/2	Checkpoint kinase1/2	SSB	single strand break
FEN1	Flap endonuclease-1	MMR	mistake match repair
TCGA	The Cancer Genome Atlas	HRR	homologous recombination repair
mPC	Metastatic prostate cancer	OR	objective response
ADPRT1	ADP ribozyme transferase-1 gene	DRD	DNA-repair gene defects
BER	Base excision repair	NER	Nucleotide excision repair
